# Two Challenges for U.S. Irrigation Due to Climate Change: Increasing Irrigated Area in Wet States and Increasing Irrigation Rates in Dry States

**DOI:** 10.1371/journal.pone.0065589

**Published:** 2013-06-05

**Authors:** Robert I. McDonald, Evan H. Girvetz

**Affiliations:** 1 Central Science Program, The Nature Conservancy, Arlington, Virginia, United States of America; 2 Global Climate Change Program, The Nature Conservancy, Seattle, Washington, United States of America; U. S. Salinity Lab, United States of America

## Abstract

Agricultural irrigation practices will likely be affected by climate change. In this paper, we use a statistical model relating observed water use by U.S. producers to the moisture deficit, and then use this statistical model to project climate changes impact on both the fraction of agricultural land irrigated and the irrigation rate (m^3^ha^−1^). Data on water withdrawals for US states (1985–2005) show that both quantities are highly positively correlated with moisture deficit (precipitation – PET). If current trends hold, climate change would increase agricultural demand for irrigation in 2090 by 4.5–21.9 million ha (B1 scenario demand: 4.5–8.7 million ha, A2 scenario demand: 9.1–21.9 million ha). Much of this new irrigated area would occur in states that currently have a wet climate and a small fraction of their agricultural land currently irrigated, posing a challenge to policymakers in states with less experience with strict regulation of agriculture water use. Moreover, most of this expansion will occur in states where current agricultural production has relatively low market value per hectare, which may make installation of irrigation uneconomical without significant changes in crops or practices by producers. Without significant increases in irrigation efficiency, climate change would also increase the average irrigation rate from 7,963 to 8,400–10,415 m^3^ha^−1^ (B1 rate: 8,400–9,145 m^3^ha^−1^, A2 rate: 9,380–10,415 m^3^ha^−1^). The irrigation rate will increase the most in states that already have dry climates and large irrigation rates, posing a challenge for water supply systems in these states. Accounting for both the increase in irrigated area and irrigation rate, total withdrawals might increase by 47.7–283.4 billion m^3^ (B1 withdrawal: 47.7–106.0 billion m^3^, A2 withdrawal: 117.4–283.4 billion m^3^). Increases in irrigation water-use efficiency, particularly by reducing the prevalence of surface irrigation, could eliminate the increase in total irrigation withdrawals in many states.

## Introduction

Anthropogenic emissions of greenhouse gases are very likely increasing average temperatures and are more likely than not altering the amount and timing of precipitation [1]. The effects of climate change on agriculture will likely be multifaceted [2]–[5]. Changes in temperature, precipitation [6] and CO_2_ concentration effects will affect plant growth. Moreover, the indirect effects of changes in weed and pest abundance and disease outbreaks may also significantly affect agriculture [7], [8]. This paper focuses on irrigation, the largest human water use, which will likely be affected by climate change [9].

Climate change will likely affect the supply of water available from surface water or groundwater, decreasing it in many cases, and may also increase evapotranspiration from crops and hence demand for irrigation [10]–[16]. While several papers have quantified how changes in climate will affect irrigation water use by agricultural producers, results have been mixed depending on the time horizon of the analysis, the particular general circulation models (GCMs) consulted, and assumptions about how producers’ irrigation practices respond to climate change [17]–[20].

The decision of whether or not to irrigate an agricultural field, as well as the amount of irrigation water applied, is a complex decision for producers, and depends on a number of factors [18], [21]. Water supply is constrained by water policy and law as well as the infrastructure available to bring water to farmers. The potential economic return from production depends on the crops grown, the productivity of the soil, and the other inputs farmers add such as fertilizer. Demand for irrigation also depends on the crop grown, as well as the prevailing climate. Climate change might increase moisture deficit, or decrease it, depending on the relative change in temperatures and precipitation. Accounting for all these factors is difficulty, which makes forecasting irrigation water use challenging.

This paper uses an empirical approach, showing for the United States that changes in climate and irrigation equipment over the past 25 years have resulted in predictable changes in both the fraction of agricultural land irrigated and the irrigation rate (m^3^ ha^−1^). We then use a simple statistical model to make state-level projections of how both quantities will be affected by climate change. We incorporate information on historical changes over time in irrigation equipment into our statistical model, and project the potential scope of future irrigation efficiency improvements to serve as an adaptation to climate change. We use this analysis to estimate the how irrigation rate and area irrigated will need to change in the future–based on the assumptions of this model–to keep up with the change in water demand of crops due to climate change.

## Materials and Methods

### Data

Data on historical withdrawals for the period 1985–2005 were obtained from the United States Geological Survey (USGS), which conducts county-level surveys every five years [22]. For this study, we use total freshwater withdrawals (surface water plus groundwater) by the agriculture sector. For our analysis, we lump county-level data to states, since the county-level data vary widely in withdrawals when the county in which an irrigation system withdraws water is different than the county where the water is applied.

Withdrawal information was supplemented by data on area irrigated, as defined in the United States Department of Agriculture (USDA) Farm and Ranch Irrigation Survey [23], and total agricultural area and its market value, as defined in the USDA Census of Agriculture [24]. In this study, our definition of “agricultural area” follows the USDA definition of “total cropland”, which includes harvested cropland, cropland used for pasture or grazing, and other miscellaneous cropland (e.g., areas where crops failed, fields were left intentionally left fallow, or with cover crops). Note that the subcategory of “cropland used for pasture and grazing” includes only land currently used for pasture or grazing that could be immediately used for crops without any additional improvement; it does not include the much larger land area of woodland and rangeland that is used for grazing or pasturing.

Data on historical temperature and precipitation were taken from the Parameter-elevation Regressions on Independent Slopes Model (PRISM) dataset (∼4 km resolution) and used to calculate historic observed moisture deficit [25]. Future climate scenarios are based on the ensemble mean of a panel of 16 general circulation models (GCM) [26], [27] for each of the A2, A1B, and B1 scenarios of the Intergovernmental Panel on Climate Change [28]. The 16 GCMs used in this study are BCCR-BCM2.0, CGCM3.1(T47), CNRM-CM3, CSIRO-Mk3.0, GFDL-CM2.0, GFDL-CM2.1, GISS-ER, INM-CM3.0, IPSL-CM4, MIROC3.2(medres), ECHO-G, ECHAM5/MPI-OM, CCSM3, PCM, and UKMO-HadCM3. For more information on the structure of each GCM, see Meehl et al. [26]. We examined three time periods: 2020–2039 (hereafter “2030”), 2040–2059 (hereafter “2050”), 2060–2079 (hereafter “2070”), and 2080–2099 (hereafter “2090”). To capture uncertainty in the GCM predictions of climate under different emissions scenarios, we also include the 20% and 80% quintiles of the ensemble’s prediction of moisture deficit.

By using an ensemble of 16 GCMs, and considering the variation among the ensemble predictions for GCMs, we hope to improve on previously published work by considering a broad set of GCMs. Other papers that have quantified how changes in climate will affect irrigation water use have only considered a few GCMs, and some of the variation in the results among and within the papers seems to be driven by the particular GCMs chosen [17]–[20]. For instance, Fisher et al. [17] looked at the climate change impacts of the a2r emissions scenario with the HadCM3 and CISRO GCMs, over the time period 1990–2080, projecting a 45% increase in irrigated area globally. Their predictions for North America varied between the two GCMs, because the CSIRO MK3 predicts a decrease in moisture deficit for much of the US, while the Hadley CM3 predicts an increase in moisture for much of the U.S. Similarly, Thomson et al. [19] looked at the climate change impacts of different average temperature increases with two GCMs, the HadCm3 and CGCM, but incorporated in their model both demand for irrigation and supply of available water. They predict that irrigated area will decline in the continental United States under both GCMs, despite strong growth in demand, because of a decline in the available supply of irrigation water.

### Calculation of Moisture Deficit

We calculated the soil moisture deficit during the growing season by subtracting precipitation from potential evapotranspiration (PET), as measured with the modified Thornthwaite (Hamon) method [29], which is based on temperature and number of daylight hours, and widely used in global and regional hydrologic models [30]. This metric should theoretically be related to irrigation water required. Moreover, it integrates intra-annual variability in water demand into a cumulative number for the growing season.

GCM predictions of moisture deficit were first resampled to average county values, and then average state values were calculated, weighting by the agricultural area (for the analysis of fraction of area irrigated) or irrigated area (for the analysis of irrigated area).

There has been considerable debate about the merits of different methods of estimating PET, and Kingston et al. [31] showed that the relative magnitude of the effect of climate change on PET depends on which method is used. The Hamon method appears to be one of the more sensitive metrics, increasing with climate change greatly, whereas other methods like Blaney-Criddle and Priestley-Taylor increase less [31]. To ensure that our results were not influence by our choice of metrics, we also calculated PET using the Blaney-Criddle method, a simple method that is known to be relatively less sensitive to climate change than most metrics [31]. The Blaney-Criddle method is a function solely of the mean daily temperature and the mean daily percentage of daytime hours.

**Figure 1:**
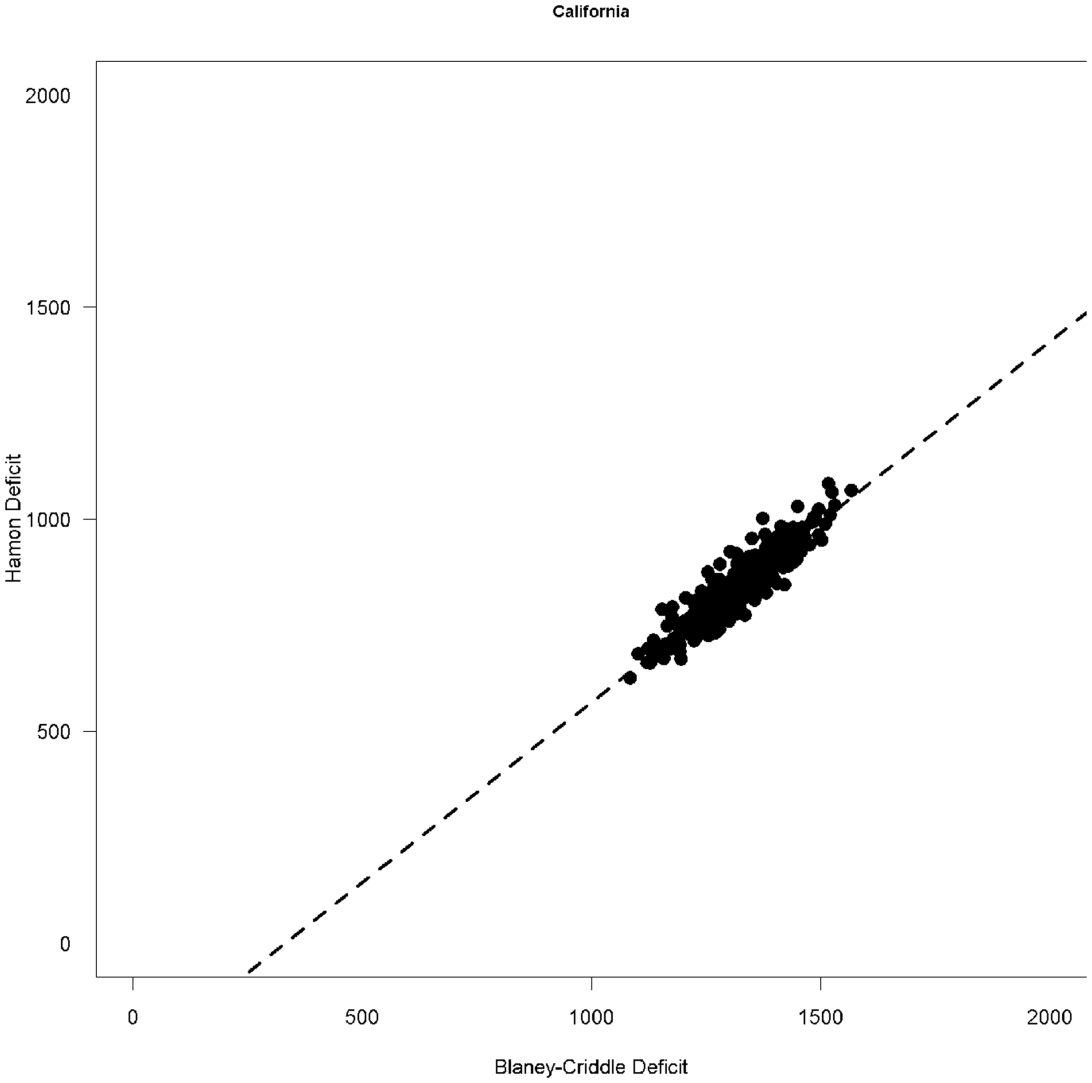
Scatterplot of estimated PET for California in 2005, using the Hamon and Blaney-Criddle metrics. Each dot represents one year in a particular combination of GCM and greenhouse gas emissions Scenario. Note that the strong linear correlation between the two means that when either of these two metrics are statistically related to irrigation use, the quantitative predictions of the effect of climate change are quite similar. Other states also show a linear correlation, with R^2^ ranging from 0.75 to 0.93.

**Figure 2:**
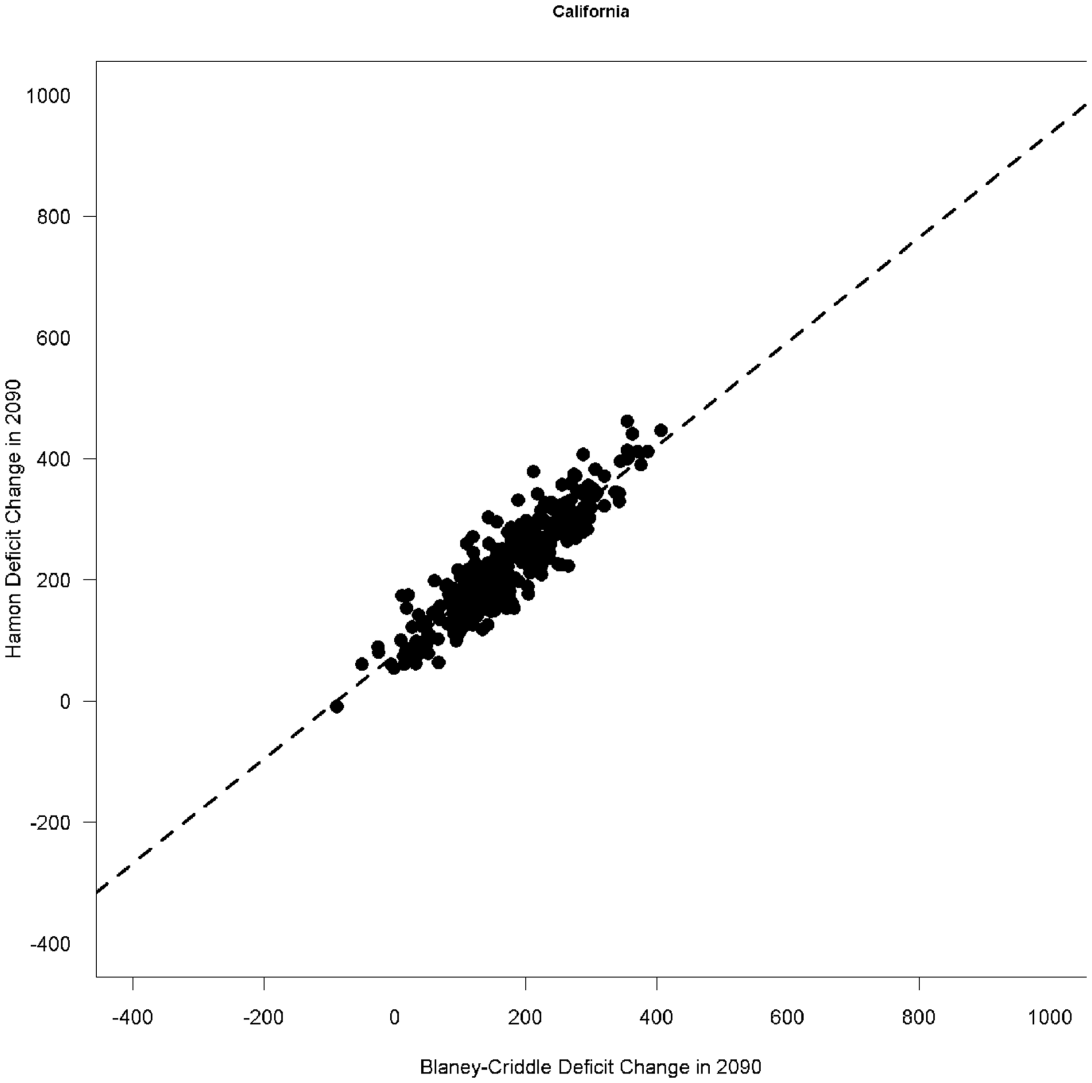
Scatterplot of change in PET for California between 2005 and 2090, using the Hamon and Blaney-Criddle metrics. Each dot represents one year in a particular combination of GCM and greenhouse gas emissions scenario. Note that the strong linear correlation between the two means that when either of these two metrics are statistically related to irrigation use, the quantitative predictions of the effect of climate change are quite similar. Other states also show a linear correlation, with R^2^ ranging from 0.74 to 0.93.

Preliminary results suggest no major change in our findings. To see why this is likely to be so, note that we build a statistical model that relates irrigation use by farmers to historical values of PET. Since Hamon and Blaney-Criddle give highly correlated estimates of PET (Figure 1), and changes in Hamon and changes in Blaney-Criddle are highly correlated (Figure 2), they both give similar results in the linear regression we use as our statistical model. While change in PET as estimated by Hamon is systematically higher than change in PET by Blaney-Criddle, the fitted slopes in our regression correct for this fact and given similar estimates of irrigation use. Because we are fitting either estimate of PET to empirical data on irrigation use, as long as the estimates are strongly linearly correlated with one another, the projections of the statistical model will be very similar.

**Figure 3:**
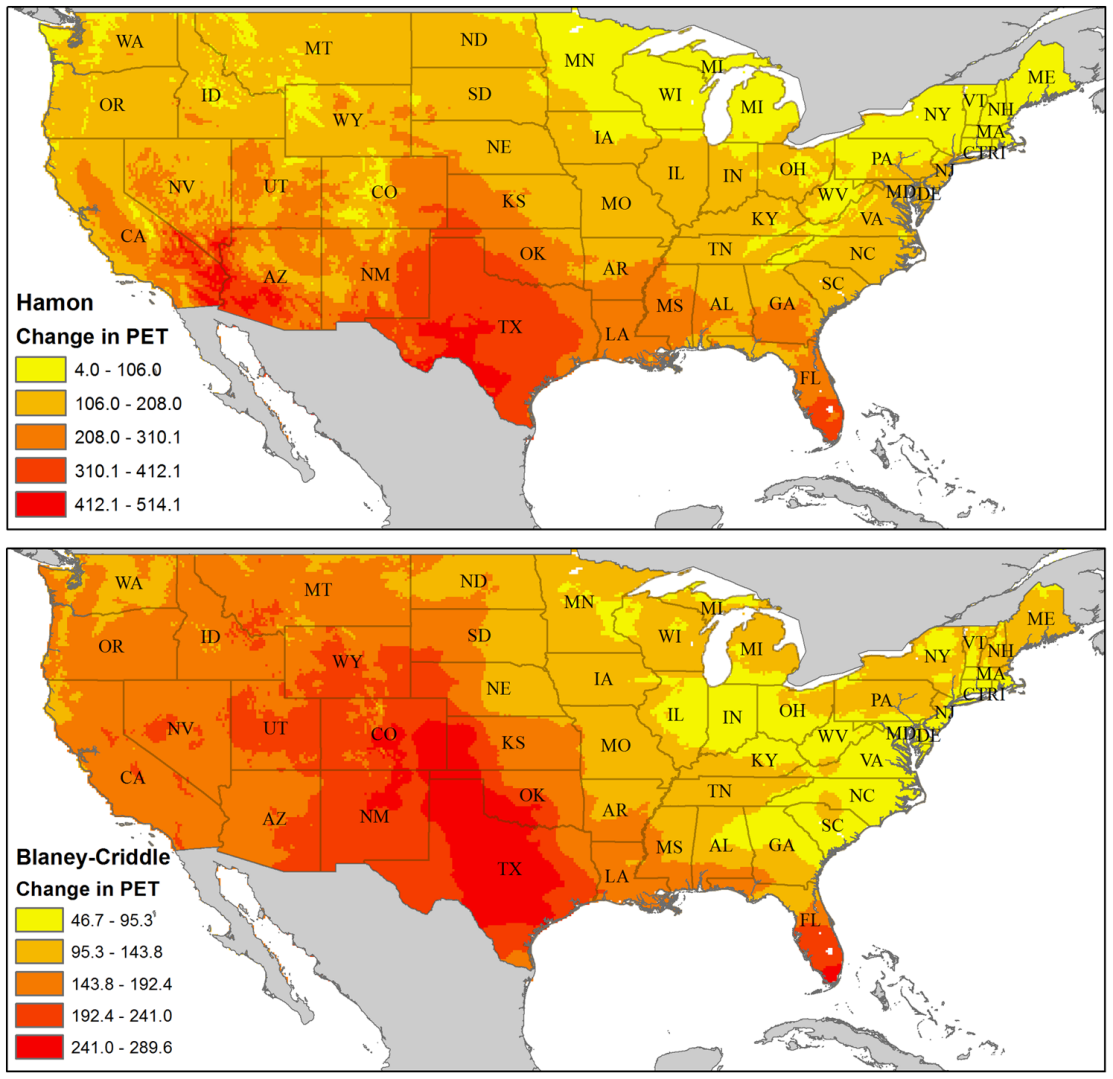
The effect of climate change on the Hamon estimate of PET (top) and the Blaney-Criddle estimate of PET (bottom). Both panels are colored with 5 equal interval categories that linearly span the range of the pixel values, with areas of less increase in PET being yellow and areas of greater increase in PET being red.

The spatial patterns of change in Hamon and Blaney-Criddle are quite similar (Figure 3). However, there are slight differences among states. For example, the Hamon metric predicts higher increases in PET in arid parts of Arizona than does Blaney-Criddle.

### Statistical Analysis

Total withdrawals were modeled as a function of total agricultural land (*A*) times the fraction of agricultural land that is irrigated (*F*) times the irrigation rate (*R*):
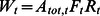



Historical information is taken from the USDA survey. *F* and *R* were both modeled as functions of moisture deficit in 1985 and the change in moisture deficit (Deficit_t_ – Deficit_1985_):
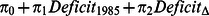
where



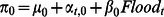


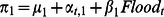


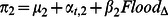



The first two terms (π_0_ and π_1_) can be interpreted as, respectively, the slope and intercept of the relationship between moisture deficit and the response variable at a point in time. This relationship is the long-term average relationship with climate, and is the product of decades of economic decisions and irrigation policies. The third term (π_2_) is the effect on the response variable of an inter-annual change in moisture deficit. It is the short term response of the agricultural sector to an interannual change in moisture deficit. All three terms are a function of a grand mean (µ), a time-specific shock (α_t_), and the percent of surface irrigation (*Flood)*. Surface irrigation (irrigation techniques where water is applied to the soil surface using gravity) was selected because it was believed to be causally negatively related to irrigation water-use efficiency. In this dataset proportion surface plus proportion sprinkler is approximately 1, since microirrigation is relatively uncommon. Note that the time-specific shock terms (α) will also incorporate other factors that varied over time but are not explicitly modeled here. For instance, if the steadily rising ambient CO_2_ concentrations increased crop water-use efficiency over the observed 20 year time period, then one would expect the α_ t,1_ to decline over time, all else being equal.

To account for autocorrelated errors by state, we used a repeated measured design for the error term, using Proc Mixed in SAS, version 9.2. We added terms one by one to the model, stopping when the added variables did not significantly improve the negative log-likelihood. The functional form of the regression of *F* was a logistic regression, although model fits using other functional forms that are bounded between 0 and 1 (e.g., probit) yielded similar results. For the regression of *R*, irrigation rates were log transformed to improve normality and to bound our results to the positive domain.

Future projections were calculated by assuming the fitted parameters in the regression equations, based on historical data, continue into the future. This implicitly assumes that some adaptation by producers takes place: regions that dry out, for example, adapt the agricultural practices of current comparable dry regions. Of course, hypothetically much larger water savings (or waste) are possible with more (or less) drastic change in agricultural practices, but we do not consider these more extreme possibilities in this analysis. Note that our analysis ignores the potential effect of increased CO_2_ concentrations increasing crop water-use efficiency, although we highlight the importance of this issue in the Discussion section.

## Results

### Analysis of Observed Historic Irrigation Patterns

**Figure 4:**
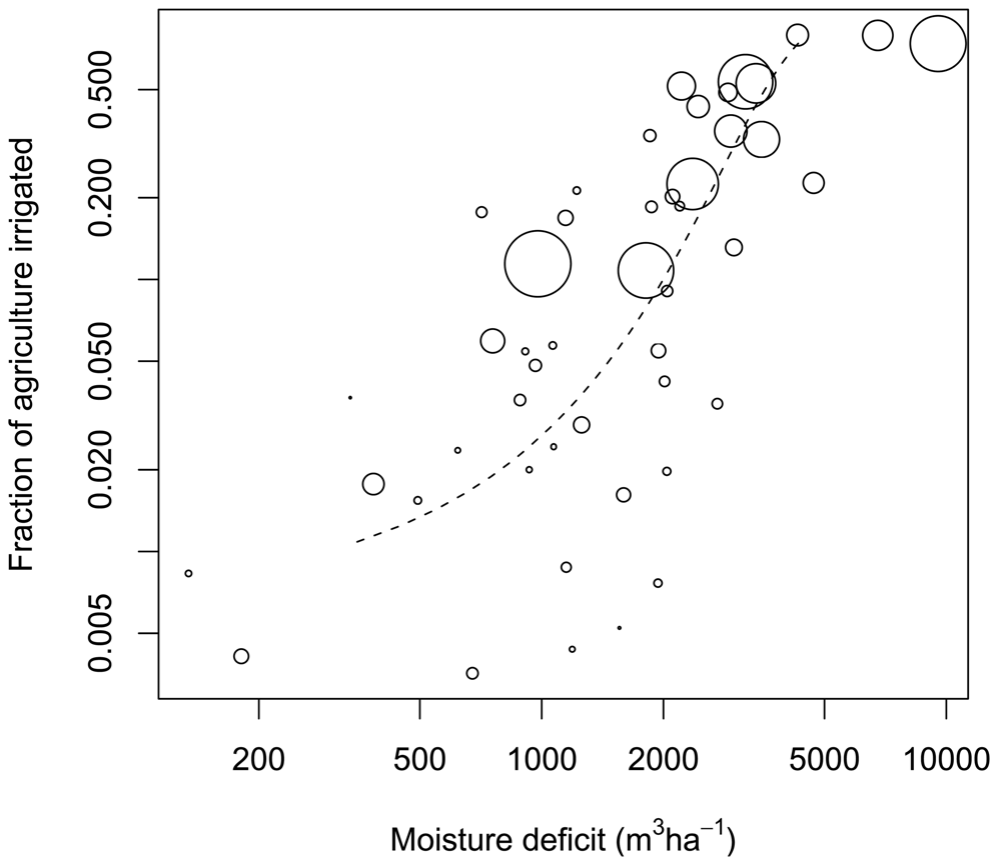
The fraction of agricultural land irrigated in U.S. states in 2005 as a function of moisture deficit. Note the logarithmic scale on both axes. The size of circle is proportional to the irrigation rate (m^3^ha^−1^); states with high moisture deficit have higher irrigation rates. The fitted regression line is shown for the middle 90% of the data range.

During 1985–2005 the proportion of irrigated agricultural land was greater in areas with greater moisture deficit (p<0.001, Table 1; and Figure 4). In any year, states with higher moisture deficits have greater proportions of agricultural land under irrigation. This relationship represents the long-term outcome of individual farmers’ decisions as a function of climate. Note that this long-term historical relationship shows adaptation to historical climate, in that dry areas are growing different crops and using different practices than wet areas. On the shorter year-to-year time scale, inter-annual changes in moisture deficit have resulted in changes to the proportion of agricultural area irrigated: higher moister deficit resulted in higher proportion of area irrigated (p = 0.03, Table 1).

**Table 1:** Regression coefficients that predict the proportion of agricultural area that is irrigated, logit transformed.

Effect	Estimate	SE	P
Intercept (*µ_0_*)	−4.4049	0.1798	<.0001
Deficit_1985_ (*µ_1_*)	0.000807	0.000067	<.0001
Deficit_Δ_ (*µ_2_*)	0.000371	0.000164	0.0252

Only the final, best fit model is shown. Greek letters correspond to the regression parameters discussed in section 2.3.

Similarly, the irrigation rate (m^3^ of water per hectare of irrigated land) is greater in sites with greater moisture deficit (p<0.001, Table 2 and Figure 4). At any point in time, states with higher moisture deficits have higher irrigation rates. Again, this relationship among different sites is the long-term outcome of a historical process of agricultural development that was influenced by the prevailing climate, and the gradient occurs despite some presumed adaptation of agricultural crops and practices to historical climate. Interestingly, the historical shift away from surface irrigation and toward sprinkler and drip irrigation has resulted in decreased irrigation rates over time (p<0.001, Table 2), all else being equal. In the short run on the year-to-year time scale, increases in moisture deficit are associated with increased irrigation rate, although this result is not statistically significant (p = 0.11, Table 2).

**Table 2:** Regression coefficients that predict the irrigation rate (m^3^ha^−1^), log transformed.

Effect	Estimate	SE	P
Intercept (*µ_0_*)	7.7495	0.07894	<.0001
Deficit_1985_ (*µ_1_*)	0.000132	0.000037	0.0009
Deficit_Δ_ (*µ_2_*)	0.000118	0.000073	0.11
Fraction Flood_1985_ *(β_1_)*	2.1475	0.4675	<.0001
Fraction Flood_Δ_ *(β_2_)*	1.3409	0.1988	<.0001

Only the final, best fit model is shown. Greek letters correspond to the regression parameters discussed in section 2.3.

### Projected Impact of Climate Change on Irrigation Area

**Figure 5:**
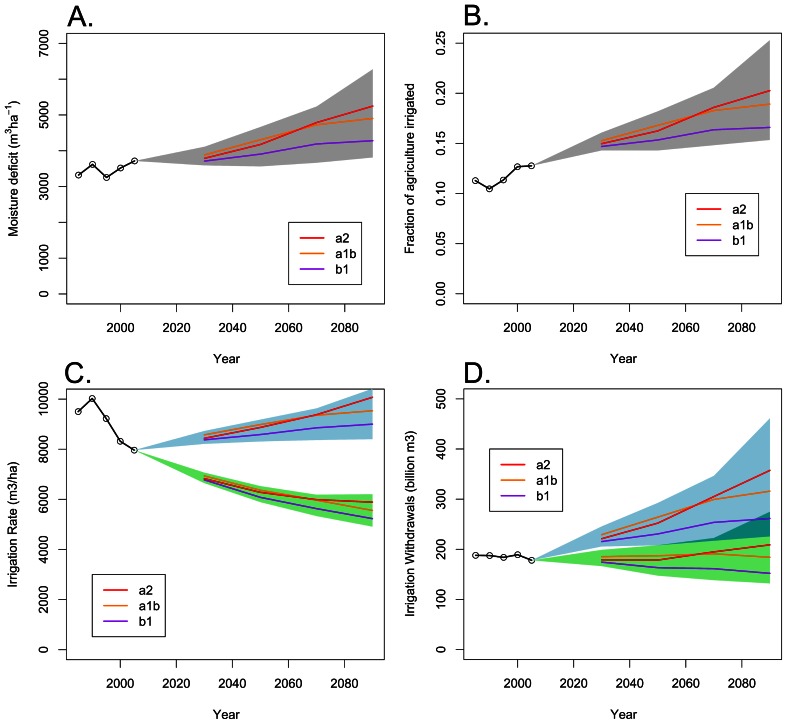
The effect of climate change on irrigation water use by United States agriculture. A.) Historical and future trends in U.S. mean moisture deficit. Displayed is the irrigated area weighted average, which is most directly relevant to irrigation rate. For each climate change scenario, the projected moisture deficit at the climate of the ensemble median is shown. The grey area shows the range of fraction irrigated for climate of the 20^th^ and 80^th^ quintiles of the ensemble. B.) Historical and future trends in the fraction of agriculture irrigated. C.) Historical and future trends in the irrigation rate. The blue area shows the effect of climate change on irrigation rates if the current mix of irrigation equipment persists over time; the green area shows the effect of climate change if the observed (1985–2005) trend away from relatively inefficient surface irrigation continues over time. D.) Historical and future trends in total irrigation withdrawals. Note the confidence intervals of the blue (current mix of irrigation equipment) and green (decreased use of surface irrigation) areas overlap after 2030.

Moisture deficit is projected to increase on average for US states under all three climate change scenarios (Figure 5A). After 2060, the increase in moisture deficit is largest in the A2 scenario, and smallest in the B1 scenario. Regardless of emissions scenario, climate change increases moisture deficit in 2090 throughout most of the contiguous US for 13 of the 16 GCMs considered [27], with the exception of the NCAR CCSM3, CSIRO MK3, and NCAR PCM models. Generally, the largest increase in moisture deficit is in the South followed by the Southwest, while the Northeast has small increases or decreases in moisture deficit. The most disagreement among climate models in the direction of moisture deficit change is in the Southwest, where some GCMs project a decrease and some an increase in moisture deficit.

Given the projected increases in moisture deficit with climate change, we project that the proportion of agricultural land irrigated in the U.S. will increase (Figure 5B). Under all emissions scenarios, farmers will increasingly need to irrigate their fields as it gets drier. The total area irrigated nationally will increase by 4.5–21.9 million ha, with the uncertainty due both to differences among emissions scenarios and to differences among GCMs. The B1 emissions scenario has the smallest increase (4.5–8.7 million ha) and the A2 emissions scenario has the greatest (9.1–21.9 million ha). Note that our projection estimates new irrigated area based on observed trends, after a moderate degree of adaptation: our methodology implicitly assumes farmers in a site made drier by climate change adopt the crops and practices of current farmers in comparable climates. Depending on the state, laws governing access to water or a shortage of available water might prevent this much expansion of irrigated area from occurring [18]. Alternatively, failure of farmers to adapt to climate change as much as is assumed in our model would imply an even larger increase in irrigated area.

**Figure 6:**
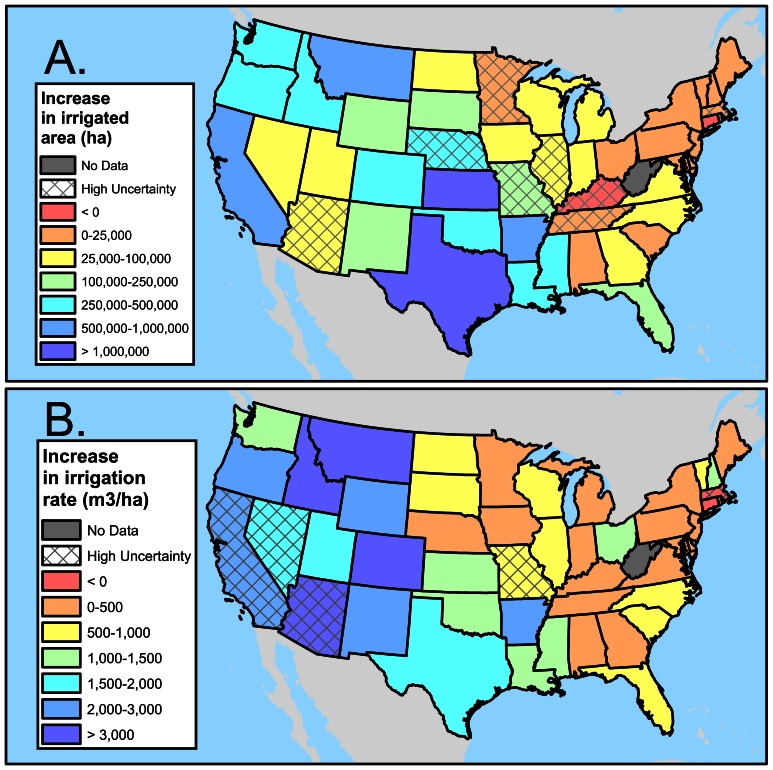
The effect of climate change on irrigation by states. A.) Projected increase in irrigated area by 2090 under the A1B scenario, ensemble median. Most states have an increase in irrigated area under all emission scenarios in more than 80% of the GCMs in the ensemble; those that have a decrease in some cases are marked high uncertainty. B.) Projected increase in irrigation rate by 2090 under the A1B scenario, ensemble median. Most states have an increase in irrigation rate under all emission scenarios in more than 80% of the GCMs in the ensemble; those that have a decrease in some cases are marked high uncertainty.

The states with the greatest demand for new irrigated area, in ha, are those in the South-Central U.S. (Figure 6A) and to a lesser extent the Northwest and California. There is projected to be less of an increase in the Northeast, where climate impacts on moisture deficit are projected to be smaller. The extent of expansion of irrigation is a function of the amount of greenhouse gas emissions, with the A2 scenario requiring the most expansion and the B1 scenario the least. The difference in projected new irrigation area demanded between the A2 scenario and the B1 scenario, at the ensemble median climate projections, is 6.4 million ha. While exact cumulative emissions depend on the GCM implementation of the scenarios, every 1 GtC increase in cumulative emissions increases the demand for new irrigated land in the US by roughly 7000 ha.

**Figure 7:**
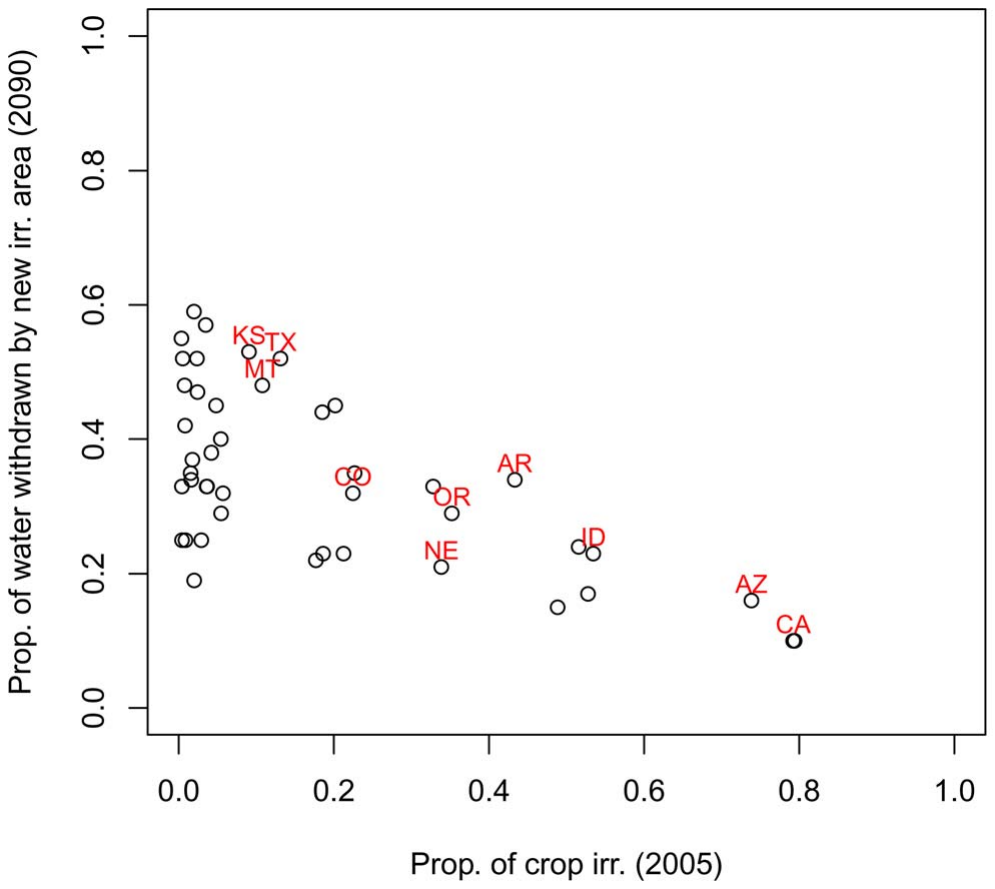
Currently wet states will have significant increases in irrigated area. The relationship between the proportion of agricultural land irrigation in 2005 and the predicted proportion of water withdrawals in 2090 (median A1B scenario of the GCM ensemble) that will come from fields not currently irrigated. A few states with significant agricultural area are labeled, using standard two-digit abbreviation for US states (see Figure 3). Three states are excluded from this graph, because climate change will have a net decrease on irrigated area there (Massachusetts, Connecticut, and Rhode Island).

This expansion of agricultural area due to climate change will pose an adaptation challenge to some states that have traditionally had a very small percentage of their agricultural land irrigation. One way to see the magnitude of this challenge is to look at the fraction of agricultural water withdrawals in 2090 that would go to fields that were not irrigated in 2005 (Figure 7). In some states with currently limited irrigation systems, more than half of all irrigation withdrawals in 2090 will be for fields that were not irrigated in 2005. Changes in Kansas (KS), Texas (TX), and Montana (MT) are particularly notable, since these states have a lot of agricultural area that is currently mostly rain-fed but will have significant expansion of their irrigation systems. Conversely, Arizona (AZ) and California (CA) already have a large fraction of their agricultural area irrigated and there is little potential for expansion to increase withdrawals in 2090.

**Figure 8:**
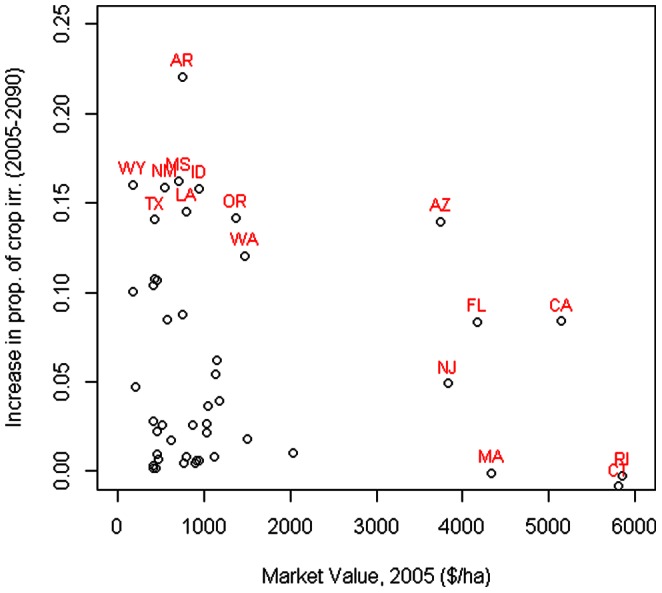
Market value and increase in proportion irrigated. The relationship between the average per hectare market value of cropland in 2005 and the predicted change in proportion of agricultural area irrigated (median A1B scenario of the GCM ensemble). A few states are labeled, using standard two-digit abbreviation for US states (see Figure 3).

Installing irrigation in new fields costs money, and producers will only install irrigation if they find the increased revenue from crops grown on irrigated fields worth more than the cost of irrigation. While a full analysis of the costs and benefits of installing irrigation is beyond the scope of the paper, some insights can be gained by looking at state-level averages (Figure 8). In some states that will have a large increase in proportion of agricultural area irrigated, current market value per ha of cropland is fairly high, and may be enough to support the costs of installing irrigation equipment. Conversely, producers in states with lower market value per ha may find it difficult to support the installation of irrigation equipment unless new crops or markets are found. For instance, both Arizona (AZ) and Texas (TX) are projected to have increased irrigated area in response to climate change. However, Arizona has a relatively high average market value for cropland of $3700 ha^−1^, driven by production of high-value crops like vegetables and horticultural products, while Texas has a relatively low average market value for cropland of $420 ha^−1^, primarily from production of grains and cotton.

### Projected Impact of Climate Change on Irrigation Rate

Increases in moisture deficit caused by climate change will likely increase the irrigation rate (Figure 5C). Under all emissions scenarios, irrigation rates will increase with climate change from 7,963 to 8,400–10,415 m^3^ha^−1^ in 2090 if the current mix of irrigation technologies stays in place. The B1 emissions scenario has the smallest rate (8,400–9,145 m^3^ha^−1^), while the A2 emissions scenario has the largest rate (9,380–10,415 m^3^ha^−1^). However, if the current trend away from surface irrigation continues at the same pace into the future, needed irrigation rates would decline from current levels to 4,911–6,205 m^3^ha^−1^ in 2090 (B1 rate: 4,911–5,355 m^3^ha^−1^, A2 rate: 5,475–6,205 m^3^ha^−1^). Note that our projection only estimates irrigation rates if current observed trends continue, after a moderate degree of adaptation: our methodology implicitly assumes farmers in newly dry climates will irrigate at a rate like that of comparable dry climates. Water policy or water availability might constrain increases in the irrigation rate. Alternatively, failure of farmers to adapt to climate change as much as is assumed in our model would imply an even larger increase in the irrigation rate.

Note also that higher CO_2_ levels might increase the water-use efficiency of crop plants, which would further lower the curves in Figure 5C. However, the degree to which this water-use efficiency increase from higher CO_2_ concentrations will occur in real systems is unclear, depending on the water balance of the site and the plant species involved [32]–[38]. For one important crop species, wheat, increases in water-use efficiency due to higher CO_2_ concentrations may exceed 20% in some circumstances [37], [39], [40]. Interestingly, the α_t,1_ terms in our model were not statistically significant and are not included in our final model, so there is no clear historical effect of rising ambient CO_2_ concentrations on crop water-use efficiency.

The second challenge that climate change poses for irrigation in the US will be this increase in irrigation rates, particularly important in already arid climates. If the current mix of irrigation technologies continues into the future, the biggest increase in irrigation rate is in the Western US (Figure 6B). In places like Arizona, California, Montana, Nevada, and Idaho, farmers will have incentive to apply more water to irrigated lands. However, in some of these states (Arizona, California, and Nevada) there is high uncertainty in the predictions of the ensemble of the GCMs (Figure 6B).

**Figure 9:**
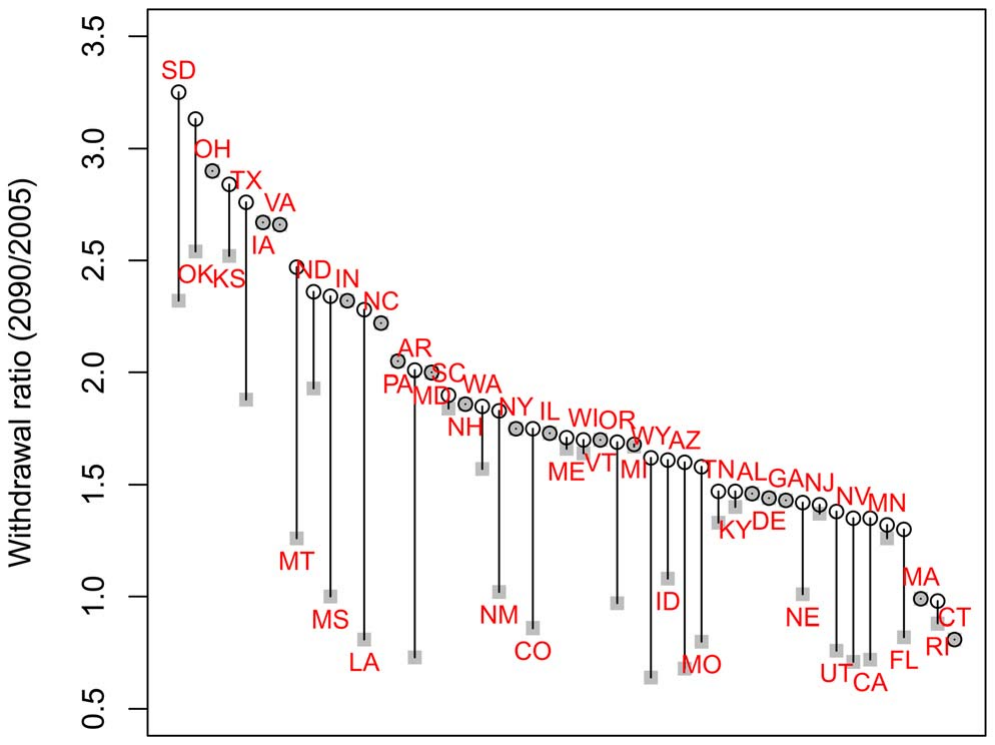
Withdrawal ratios by state. The ratio of irrigation withdrawals in 2090 to irrigation withdrawals in 2005 if the mix of irrigation technologies stays the same as today (open circles) or if the current trend away from surface irrigation continues into the future (grey squares). A ratio of more than one indicates withdrawals will increase, while a value of less than one indicates withdrawals will decrease. Data points are labeled using the standard two-digit abbreviation for US states (see Figure 3), staggered so that labels do not overlap.

Moving away from surface irrigation could generate substantial irrigation water-use efficiency gains, offsetting demand for an increase in irrigation rate in these states (Figure 9). States with the greatest potential for decrease in irrigation rate with a move away from surface irrigation are Arkansas (AR), Louisiana (LA), Arizona (AZ), Wyoming (WY), and Colorado (CO). In these states, the potential for decreased irrigation rate can more than offset the potential for irrigated area expansion due to climate change. On the other hand, some states such as Iowa and Illinois currently use very little surface irrigation, and there is little potential for decreasing irrigation rates with a move away from surface irrigation. Note also that there is considerable variability among GCMs for the Southwest, and while the median projection under all emissions scenarios is a large increase in irrigation rates in these states, a few GCMs actually project a decrease in moisture deficit and hence a decrease in irrigation rates in these states.

In the absence of gains in irrigation water-use efficiency, total water withdrawals for irrigation are likely to increase substantially (Figure 5D). This is true over all emissions pathways, but the increase in particularly striking for the A2 emissions pathway. Thus, irrigation water-use efficiency gains from switching away from surface irrigation can be an effective adaptation under moderate climate change, but cannot be wholly effective if climate change is severe. Moreover, increases in irrigation efficiency can only serve as adaptation effectively in some states (Figure 9).

## Discussion

Our results suggest that there are two different challenges that climate change will pose to agricultural irrigation in the US. In states in currently relatively wet climates, expansion of irrigated area is likely to be an important driver in increases in overall agricultural withdrawals. In some cases, these states have relatively little experience managing water use under scarcity, at least compared to states in the western US. For instance, many states in the eastern United States use a riparian water rights system, where all land owners along a waterbody have a right to make reasonable use of water at any time. In these states, there may currently be little regulation on the installation of a new irrigation system. Our results suggest that a key goal for these states should be developing appropriate regulations that make sure new irrigation systems have access to sufficient water without affect other water users in the area.

In contrast, in states in currently dry climate that already have a large fraction of agricultural area irrigated, our results project the predominant driver of increases in water withdrawal will be the tendency of producers to apply more irrigation water in response to droughts. Fully using technologies to increase irrigation efficiency will be a key goal for these states. The switch away from surface irrigation is included in our model, but many other forms of adaptation are possible [2], [41]. Changes in agricultural practices such as tilling and plant spacing can affect water demand, as can changes in crop varieties beyond what is implicitly assumed by our model. Many of these currently dry states are in the western US and follow a prior appropriation water rights system, where older uses of water have priority over new uses of water. New users of water may be limited by their water right in how much they can increase their irrigation volume. However, currently many states lack mechanisms for water shortages to affect the price or distribution of existing water rights to farmers, leaving them little incentive to consider low water availability in their individual decision-making [9], [42]–[45]. Farmers would have more incentive to switch to more water-efficient crops if the price of irrigation water more accurately reflects its scarcity.

Our results also highlight the importance of greenhouse gas mitigation for reducing impacts on freshwater demand. The median difference in extra demand for withdrawals in the A2 and B1 scenarios is 57 billion m^3^ per year in 2090. This means that avoiding the release of 1 GtC will result in the U.S. needing roughly 65 million m^3^ less irrigation water annually. Our results demonstrate that, in addition to reducing climate change’s many other impacts, climate change mitigation saves irrigation water.

Our results stress that climate change will likely increase irrigation water use in the US, consistent with some previously published research [17], [18], [21], but in contrast with the results of other studies that showed mixed results depending on the GCM examined [19], [20]. At the same time, other research suggests climate change will decrease water supply, at least in some regions, either because of changes in annual supply or because of intra-annual changes in the hydrologic cycle such as changes in snowpack [19]. The exact response of agricultural producers depends on irrigation policy and law, the price of irrigation and water, and the income from crop production, among other things.

The central assumption when making climate change projections with such a statistical model is that past is prologue: how agricultural irrigation changed in response to past climate events is indicative of how it will respond in the future. While this is a reasonable assumption used by many modeling studies, readers should remember that more radical changes in water governance or water availability in the U.S. are theoretically possible, and would imply different forecasts than the ones we present.

Further research into the mechanisms that control the decisions of agricultural producers is key to evaluating the extent to which observed trends in the past two decades are indicative of the response of the agricultural system climate change. For instance, additional field-based studies of the impact of increased CO_2_ on crop water use efficiency are needed to incorporate these feedbacks into mechanistic models of responses to climate change. Other factors known to affect irrigation withdrawals include the price of irrigation and income from commodity production.

Our simple statistical model provides a useful complement to more complex, mechanistic models of agricultural irrigation use. This model points out on a national scale how climate change is projected to impact agricultural water demand in different states, and how those states will likely be driven to change their agricultural practices in response to these impacts–either through increasing the area of agricultural land irrigated or through increasing the irrigation rate on already irrigated land. This information is useful to agricultural and water resource managers for informing their long-term planning, and provides insight more broadly into how changes to agricultural water demand will likely complicate water management in the future, among many other demands from other sectors.
